# MTAP-ANRIL gene fusion promotes melanoma epithelial-mesenchymal transition-like process by activating the JNK and p38 signaling pathways

**DOI:** 10.1038/s41598-023-36404-w

**Published:** 2023-06-05

**Authors:** Zhuoying Lin, Yu Lei, Mingyao Wen, Qin He, Dean Tian, Huaping Xie

**Affiliations:** 1 Department of Gastroenterology, Shangrao People’s Hospital, Shangrao, 334000 Jiangxi Province China; 2 Department of Gastroenterology, Tongji Hospital of Tongji Medical College, Huazhong University of Science and Technology, Wuhan, 430030 Hubei Province China; 3 Institute of Liver and Gastrointestinal Diseases, Huazhong University of Science and Technology, Tongji Hospital of Tongji Medical CollegeWuhan, 430030 Hubei Province China; 4 Department of Otolaryngology-Head and Neck Surgery, Tongji Hospital of Tongji Medical College, Huazhong University of Science and Technology, Wuhan, 430030 Hubei Province China

**Keywords:** Metastasis, Skin cancer

## Abstract

Gene fusions caused by cytogenetic aberrations play important roles in the initiation and progression of cancers. The recurrent MTAP-ANRIL fusion gene was reported to have a frequency of greater than 7% in melanoma in our previous study. However, its functions remain unclear. Truncated MTAP proteins resulting from point mutations in the last three exons of MTAP can physically interact with the wild-type MTAP protein, a tumor suppressor in several human cancers. Similarly, MTAP-ANRIL, which is translated into a truncated MTAP protein, would influence wild-type MTAP to act as an oncogene. Here, we found that MTAP-ANRIL gene fusion downregulated the expression of wild-type MTAP and promoted epithelial-mesenchymal transition-like process through the activation of JNK and p38 MAPKs in vitro and in vivo. Our results suggest that MTAP-ANRIL is a potential molecular prognostic biomarker and therapeutic target for melanoma.

## Introduction

Melanoma is one of the most aggressive malignancies and is prone to metastasize in a subset of patients. In 2018, 287,723 new cases of cutaneous melanoma were diagnosed worldwide, accounting for 1.6% of primary malignancies (excluding nonmelanoma skin cancer), and 60,712 cancer deaths (0.6% of all cancer deaths) were attributed to cutaneous melanoma^1^. Once it metastasizes, melanoma is life-threatening, and few available treatments are successful^2,3^. The poor prognosis of metastatic melanoma requires the development of a better understanding of the molecular mechanism driving the malignant phenotype.

Chromosomal abnormalities, such as translocations, inversions, insertions and deletions, probably lead to gene fusions due to the exchange of coding or regulatory DNA sequences between genes^4^. Structural chromosomal rearrangements, including gene fusions, have been reported to trigger or maintain predominantly hematological disorders and mesenchymal tumors rather than epithelial tumors. However, based on large amounts of cytogenetic data in 44,750 neoplasms containing hematological malignancies and solid tumors, no fundamental tissue-specific differences were found in the genetic mechanisms of neoplastic initiation. Therefore, cytogenetic alterations may also play an important role in the initial step of epithelial tumorigenesis^5^. Recurrent gene fusions between TMPRSS2 and ETS family genes were first detected in prostate cancer and proven to drive the development as well as the progression of most prostate cancers^6,7^. Subsequently, several other recurrent gene fusions were discovered in different kinds of epithelial tumors, such as those of lung, stomach, breast and colorectal cancers^8–13^. In melanoma, only sporadic cases with gene fusions have been reported. For instance, one case with BRAF and RAF1 rearrangement was detected among 131 melanoma cases^14^, and six cases with MET-related gene fusions were reported among 1202 melanocytic neoplasms^15^. Even among 333 cutaneous melanomas from The Cancer Genome Atlas (TCGA) Network, recurrent gene fusions were rare among the 224 gene fusions detected.

Chromosome 9p21 contains the CDKN2A/2B locus, which encodes tumor suppressor genes that are inactivated via mutation, deletion and promoter methylation in a variety of human cancers, especially melanoma^16,17^. Based on fine mapping of chromosome 9p21 deletions in melanoma cell lines, recurrent gene fusions between methylthioadenosine phosphorylase (MTAP) and antisense noncoding RNA in the INK4 locus (ANRIL)—namely, MTAP-ANRIL gene fusions—were reported in our previous study^18^. The MTAP-ANRIL fusion gene is the most frequently identified fusion gene in melanoma, with a frequency of greater than 7% across all melanoma cell lines and approximately 20% in melanoma cell lines and tissues with chromosome 9p21 deletion^18^. Recently, MTAP-ANRIL fusion gene was also confirmed in acute lymphoblastic leukemia, with a frequency of 5% (15/279) in acute lymphoblastic leukemia^19^. However, the function of this fusion gene remains unclear.

MTAP is a ubiquitously expressed homotrimeric subunit enzyme in the methionine salvage pathway that metabolizes the byproduct of polyamine synthesis, 5′-methylthioadenosine (MTA), leading to eventual regeneration of methionine and adenine^20^. Studies have shown that MTAP acts as a suppressor gene and inhibits the proliferation, migration and invasion of tumor cells^21,22^. MTAP deletion reduces the methyltransferase activity of protein arginine methyltransferase 5 (PRMT5), increases the sensitivity to PRMT5 depletion and confers selective dependence on PRMT5 and its binding partner WDR77 in cancer cells; therefore, inhibitors of PRMT5 are a potential therapeutic target in MTAP-deleted tumors^23–25^. In addition, loss of MTAP results in epithelial-mesenchymal transition (EMT) via the GSK3β/Slug/E-cadherin axis in esophageal squamous cell carcinoma cells^26^. ANRIL regulates target genes, leading to increased cell proliferation, increased cell adhesion and decreased apoptosis^27^. Similar to the results in our previous study, point mutations in the last three exons of MTAP were found to result in six truncated MTAP isoforms, each of which could physically interact with wild-type (WT) MTAP and cause hereditary malignant fibrous histiocytoma^20,28^. Quite possibly, MTAP-ANRIL gene fusions play a role in melanoma metastasis by acting on wild-type MTAP. In our present study, we found that MTAP-ANRIL gene fusion downregulated the expression of wild-type MTAP and promoted EMT-like process through the activation of JNK and p38 MAPKs in vitro and in vivo.

## Materials and methods

### Cell lines

Human melanoma cell lines (A375 and A875) were kindly donated by the Department of Dermatology, Tongji Hospital, Tongji Medical College, Huazhong University of Science and Technology. Cells were cultured in Dulbecco’s modified Eagle’s medium (DMEM) supplemented with 10% FBS, 100 μg/ml penicillin and 100 μg/ml streptomycin at 37 °C in a 5% CO2 incubator.

### RT-PCR

Total RNA was extracted using TRIzol Reagent (Takara, Japan), and reverse transcription was performed using an Advantage RT-for-PCR Kit (Vazyme, China) according to the manufacturer’s instructions. For real-time PCR analyses, aliquots of double-stranded cDNA were amplified using a SYBR Green PCR Kit (Vazyme, China). The cycling parameters were as follows: 95 °C for 15 s, 60 °C for 15 s, and 72 °C for 15 s for 45 cycles. The primer sequences were as follows: β-Actin: 5′-CATGTACGTTGCTATCCAGGC-3′ (sense) and 5′-CTCCTTAATGTCACGCACGAT-3′ (antisense); MTAP-ANRIL: 5′-GGAGCACGAGGAAGCATGTC-3′ (sense) and 5′-GCAACTAGAAGGCACAGTCG-3′ (antisense); CDH1(E-cadherin): 5′-AAAGGCCCATTTCCTAAAAACCT-3′ (sense) and 5′-TGCGTTCTCTATCCAGAGGCT-3′ (antisense); CDH2(N-cadherin): 5′-TCAGGCGTCTGTAGAGGCTT-3′ (sense) and 5′-ATGCACATCCTTCGATAAGACTG-3′ (antisense); Cyclin B1: 5′-TTGGGGACATTGGTAACAAAGTC-3′ (sense) and 5′- ATAGGCTCAGGCGAAAGTTTTT-3′ (antisense); Cyclin D1: 5′- GCTGCGAAGTGGAAACCATC-3′ (sense) and 5′-CCTCCTTCTGCACACATTTGAA-3′ (antisense); Snail1: 5′-ACTGCAACAAGGAATACCTCAG-3′ (sense) and 5′-GCACTGGTACTTCTTGACATCTG-3′ (antisense); Snail2: 5′-TGTGACAAGGAATATGTGAGCC-3′ (sense) and 5′-TGAGCCCTCAGATTTGACCTG-3′ (antisense). Relative gene expression levels were calculated using the formula 2(− ΔΔCt). All experiments were performed in triplicate.

### Plasmid construction

Plasmid construction was performed according to standard procedures as outlined in http://www.genechem.com.cn. This construct corresponds to the sequence from the MTAP-ANRIL fusion gene (KT386340.1). The polymerase chain reaction (PCR) product was cloned into the Nhel and BamHl sites of the pcDNA3.1(+) vector with FLag. And the forward primer of the target gene sequence is 5′-ACGGGCCCTCTAGACTCGAGCGCCACCATGGCCTCTGGCACCACCAC-3′, the reverse primer is 5′-AGTCCAGTGTGGTGGAATTCTCAAAAGGGACATGCTTCCTC-3′.

### The establishment of stable expressing cells

For establishment of stable expressing cells, plasmids (Genechem Company, China) were transfected into cells with Lipofectamine 2000 according to the manufacturer’s instructions. We achieved the stable overexpression transfectant by adding G418 (Sigma Aldrich) for 4 weeks.

### Western blot analyses

Proteins extracted from lysed cells were separated by SDS-PAGE and transferred to nitrocellulose membranes. Nonspecific binding sites were blocked with 5% BSA in TBST (120 mM Tris-HCl (pH 7.4), 150 mM NaCl, and 0.05% Tween 20) for 1 h at room temperature. Membranes were probed with primary antibodies against FLAG (Proteintech, 20543-1-AP), MTAP (Cell Signaling Technology, #4158), E-cadherin (Proteintech, 20874-1-AP), N-cadherin (Proteintech, 22018-1-AP), Cyclin B1 (Proteintech, 55004-1-AP), Cyclin D1 (Proteintech, 26939-1-AP), total-JNK (Abcam, ab179461), phosphor-JNK (Abcam, ab124956), total-p38 (Cell signaling technology, #9212S), phosphor-p38 (Cell signaling technology, #9211), total-Akt (Cell signaling technology, #4685), phosphor-Akt (Cell signaling technology, #4060), total-ERK (Cell Signaling Technology, #9102), phosphor-ERK (Cell Signaling Technology, #4370), overnight at 4 °C. Membranes were then washed three times with TBST and incubated with HRP-conjugated secondary antibodies. Immunoreactions were detected using ImmobilonTM Western Chemiluminescent HRP substrate (Tanon, China). All experiments were performed in triplicate.

### In vitro migration and invasion assays

The migratory and invasion abilities of cells were determined using 24-well transwell plates (8 µm pore size, Corning, USA). For transwell migration assays, 5 × 10^4^ cells were plated in the upper chambers, which were lined with a noncoated membrane. After 24 h of routine culture in 37 ℃ incubators, chambers were taken out and the cells were stained after fixation. For invasion assays, the chamber inserts were coated with 200 mg/ml Matrigel and dried overnight under sterile conditions. Then, 1 × 10^5^ cells were plated in the upper chambers. The next steps are the same as the migration assays above. Images were acquired in five random fields under a microscope. All experiments were performed in triplicate.

### CCK8 assays

The proliferation of melanoma cells in vitro was measured using CCK8 assays following the manufacturer’s instructions. Briefly, 5 × 103 cells/well were seeded in 96‐well plates. At the defined time points, cells were treated with 10 μl of CCK8 solution and incubated at 37 °C for 2 h. The medium was then replaced with 100 μl of DMSO and incubated at room temperature for 10 min with shaking. Optical densities were measured spectrophotometrically at a wavelength of 450 nm. All experiments were performed in triplicate.

### In vivo tumor growth assays

Suspensions of transfected A875 cells were prepared using cell culture medium, and the concentration was adjusted to 1 × 106 cells/μl. A 100 μl volume of the cell suspension was inoculated subcutaneously into mice. Tumor formation in the nude mice was monitored over a 30 days period. The tumors were measured every 3 days, and the tumor volumes were calculated as 1/2 (largest diameter) × (smallest diameter)2.

### In vivo metastasis assays

For metastasis assays, 1 × 106 A875 cells in 100 μl of phosphate-buffered saline (PBS) were injected into the tail vein of nude mice. Mice were sacrificed on day 30 by neck-breaking execution. Lung tissues were resected, fixed with 4% paraformaldehyde and stained with H&E.

### Quantification and statistical analysis

All values were recorded as the mean ± standard deviation (sd). *P* values were statistically analyzed by the χ^2^ test for categorical variables and by Student’s test for quantitative data. Statistical values were calculated with SPSS software (Version 20.0).

### Ethics approval and consent to participate

In this study, all experiments on live vertebrates were approved by animal ethics management committee of Tongji Hospital. And all experiments were performed in accordance with relevant guidelines and regulations.

## Results

### MTAP-ANRIL promoted melanoma cell migration, invasion and proliferation in vitro

**Figure 1:**
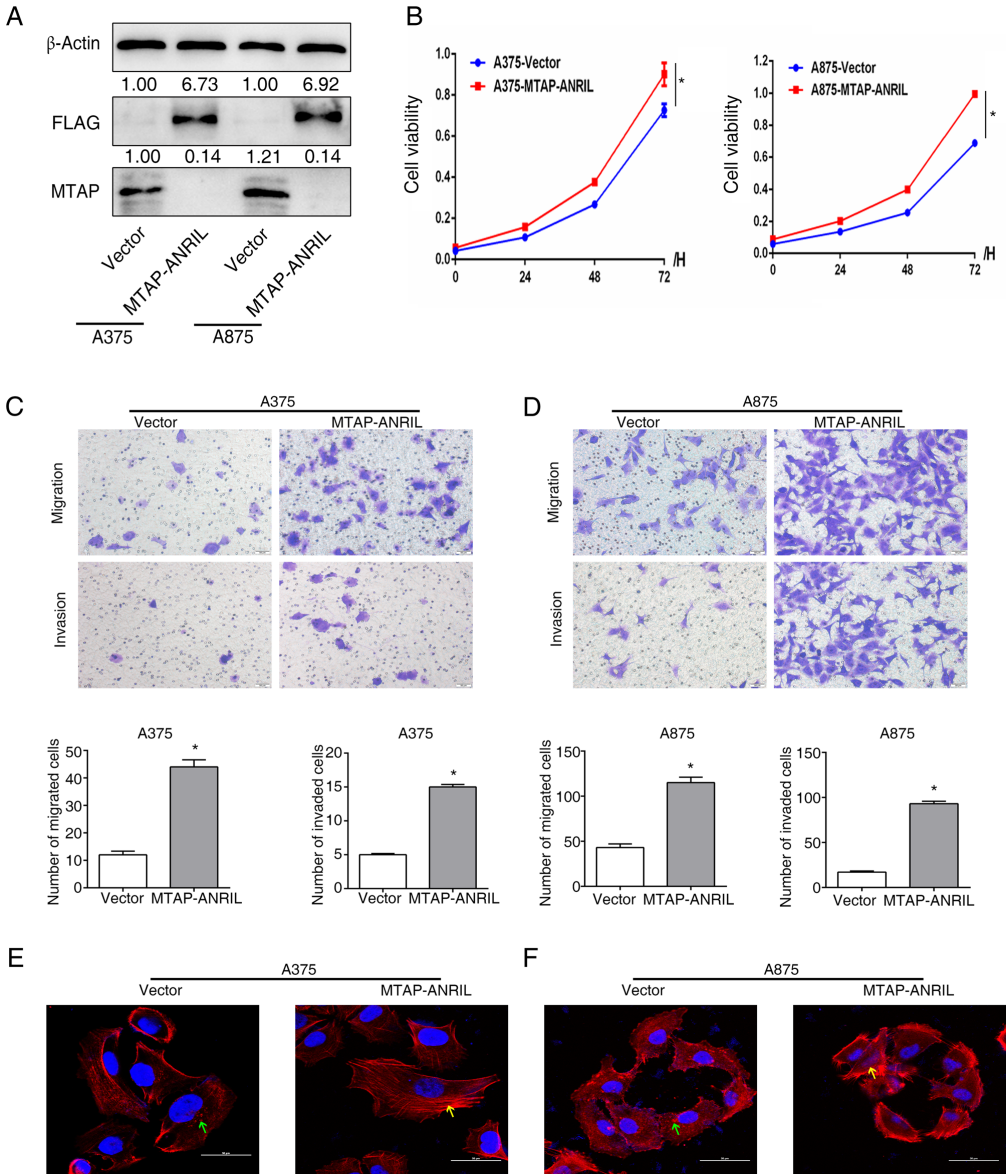
Overexpression of MTAP-ANRIL promoted melanoma migration, invasion and proliferation in vitro. (**A**) The MTAP-ANRIL fusion gene plasmid with Flag was transinfected into A375 and A875 cells. Western blot analysis showed that the expression of Flag which represented the plasmid was successfully transinfected into cells, and the expression of MTAP which was truncated protein after transfecting the MTAP-ANRIL plasmid. (**B**) CCK8 assays indicated that overexpression of MTAP-ANRIL promoted the cell viability of A375 (left) and A875 (right) melanoma cells. (**C**, **D**) Representative cell images and quantifications from transwell migration and invasion assays with A375 cells (**C**) and A875 cells (**D**) overexpressing the MTAP-ANRIL fusion gene. (**E**, **F**) MTAP-ANRIL overexpression diminished the epithelial phenotype of A375 (**E**) and A875 (**F**) melanoma cells. F-actin cytoskeletal arrangement was examined by fluorescence microscopy in melanoma cells. Green arrows: punctate F-actin, yellow arrows: cortical F-actin organized as a curvilinear network. The data are presented as the mean ± SD values. *P < 0.01.

To investigate the function of the MTAP-ANRIL fusion gene, we constructed a MTAP-ANRIL fusion gene plasmid and transfected it into melanoma cell lines to establish the A375-MTAP-ANRIL and A875-MTAP-ANRIL stable cell lines (Fig. 1A). To verify whether the MTAP-ANRIL fusion gene is important for cell viability, the cell viability was assessed by CCK8 assays. Overexpression of MTAP-ANRIL significantly increased melanoma cell proliferation (Fig. 1B). We used a transwell assay to evaluate the migration and invasion abilities of melanoma cells overexpressing MTAP-ANRIL compared with control cells. MTAP-ANRIL overexpression enhanced the cell migration and invasion abilities compared with those of control cells (Fig. 1C,D). Furthermore, MTAP-ANRIL overexpression decreased E-cadherin expression but increased N-cadherin expression, indicating that MTAP-ANRIL promoted EMT-like process (Fig. 3A,B). To explore whether MTAP-ANRIL could bind to the cytoskeleton of melanoma cells, the effect of MTAP-ANRIL on F-actin cytoskeletal arrangement was assessed by immunofluorescence microscopy. Although punctate F-actin was observed in control cells, F-actin fibers were densely arranged and formed circular bundles in the cytoplasm of MTAP-ANRIL-overexpressing cells (Fig. 1E,F). This organization is characteristic of cells with apical-basolateral polarity, such as epithelial cells.

### MTAP-ANRIL promoted melanoma cell migration, invasion and proliferation in vivo

**Figure 2:**
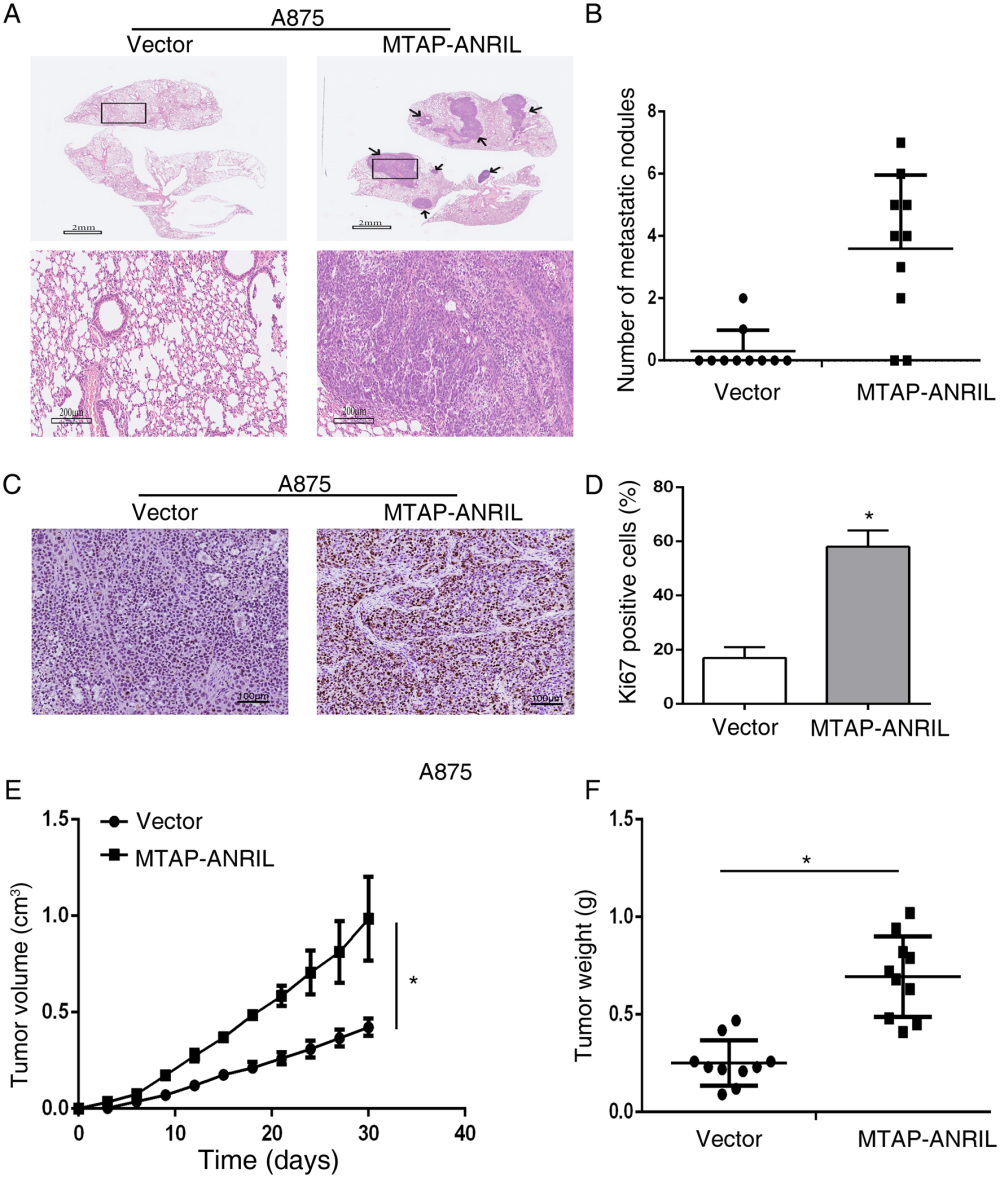
Overexpression of MTAP-ANRIL promoted metastasis and proliferation in vivo. (**A**) Representative hematoxylin and eosin (H&E) staining images of lung samples from mice injected with A875 melanoma cells. The black arrows indicate the lung metastatic tumors. (**B**) The number of metastatic nodules in the lungs of mice injected with A875 melanoma cells was analyzed. (**C**, **D**) Representative Ki67 staining images of tumors from mice injected with A875 melanoma cells. (**E**, **F**) The volume and weight of tumors in mice injected with A875 melanoma cells were calculated. The data are presented as the mean ± SD values. *P < 0.01.

Mice injected with A875-MTAP-ANRIL cells showed more lung metastases than those injected with vector-expressing cells (Fig. 2A,B). Moreover, MTAP-ANRIL overexpression increased tumor cell proliferation, as indicated by the increase in ki67-positive cells (Fig. 2C,D), and significantly promoted tumor growth (Fig. 2E,F). Collectively, these findings implied that the MTAP-ANRIL fusion gene promoted melanoma cell migration, invasion and proliferation.

### The MTAP-ANRIL fusion gene regulated melanoma migration, invasion and proliferation-related genes

**Figure 3:**
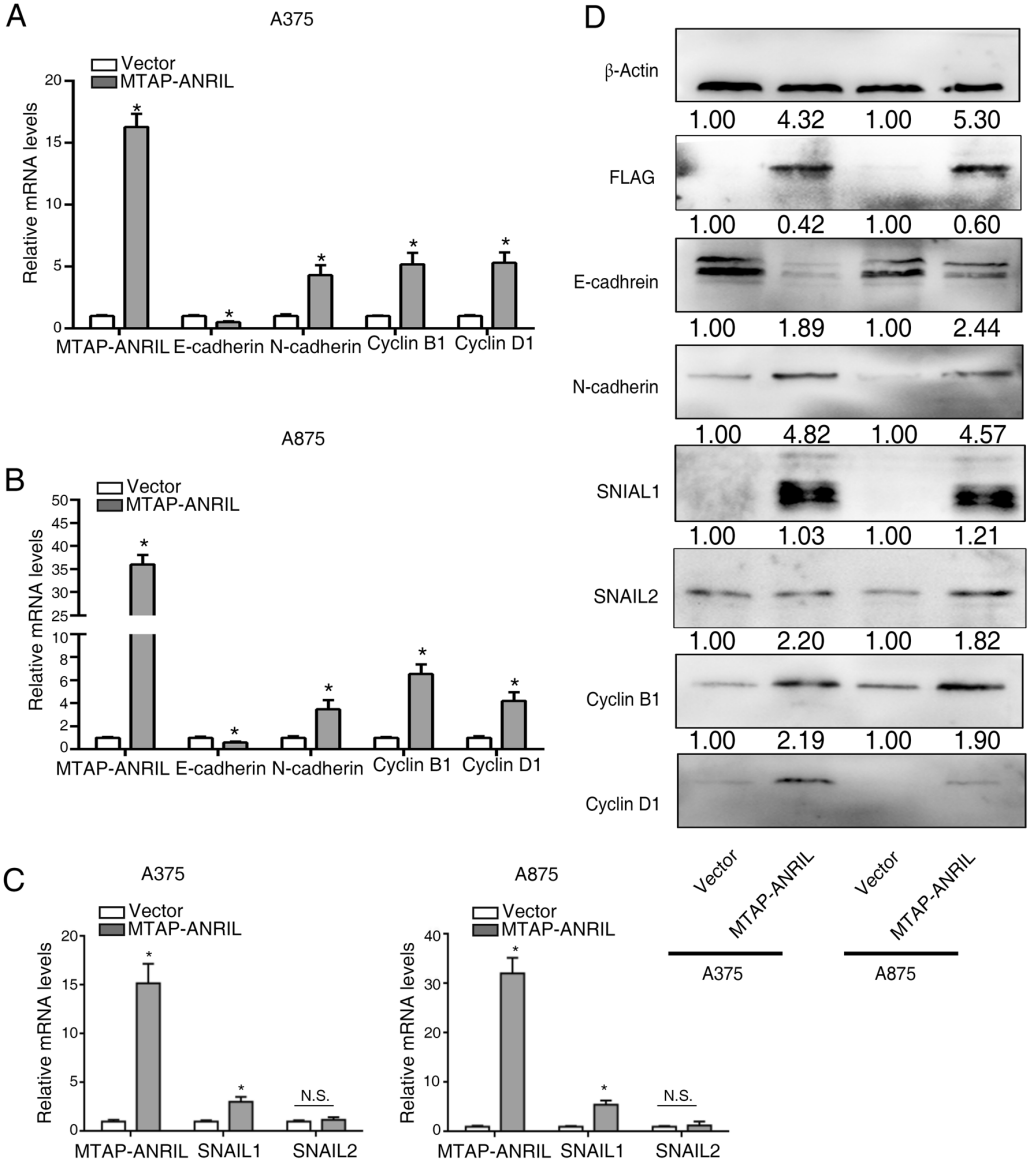
Overexpression of MTAP-ANRIL regulated the expression of metastasis and proliferation-related genes. (**A**, **B**) Real-time PCR analysis of metastasis-related gene (E-cadherin and N-cadherin) expression and proliferation-related gene (Cyclin B1 and Cyclin D1) expression in A375 (**A**) and A875 (**B**) melanoma cells after transfection with the MTAP-ANRIL plasmid. (**C**) Real-time PCR analysis of EMT-related gene (SNAIL1 and SNAIL2) expression in A375 (left) and A875 (right) melanoma cells after transfection with the MTAP-ANRIL plasmid. (**D**) Western blot analysis of metastasis-related gene (E-cadherin and N-cadherin) expression, EMT-related gene (SNAIL1 and SNAIL2) and proliferation-related gene (Cyclin B1 and Cyclin D1) expression in melanoma cells after transfection with the MTAP-ANRIL plasmid. The data are presented as the mean ± SD values. *P < 0.01.

MTAP deletion is involved in EMT21, and MTAP as well as ANRIL are related to cell migration, invasion and cell viability 18–19, 22. We found that MTAP-ANRIL overexpression in melanoma cells inhibited the expression of MTAP and decreased the expression of E-cadherin but increased the expression of N-cadherin, important markers of EMT. In addition, MTAP-ANRIL overexpression increased the expression of both Cyclin B1 and Cyclin D1, which are related to proliferation (Fig. 3A,B). Our results showed that MTAP-ANRIL fusion gene regulated melanoma migration and invasion abilities via EMT-like process, but the EMT transcriptional factors which involved in the experimental model is unclear. We detected the expression of SNAIL1 and SNAIL2, which are the important EMT transcriptional factors. The results showed that MTAP-ANRIL increased SNAIL1 expression, but not affect the expression of SNAIL2 (Fig. 3C). Consistently, the protein level of E-cadherin, N-cadherin, Cyclin B1, Cyclin D1, SNAIL1 and SNAIL2 had the similar results with the mRNA levels (Fig. 3D).

### MTAP-ANRIL promoted melanoma cell migration, invasion and proliferation by activating the JNK and p38 signaling pathways

**Figure 4:**
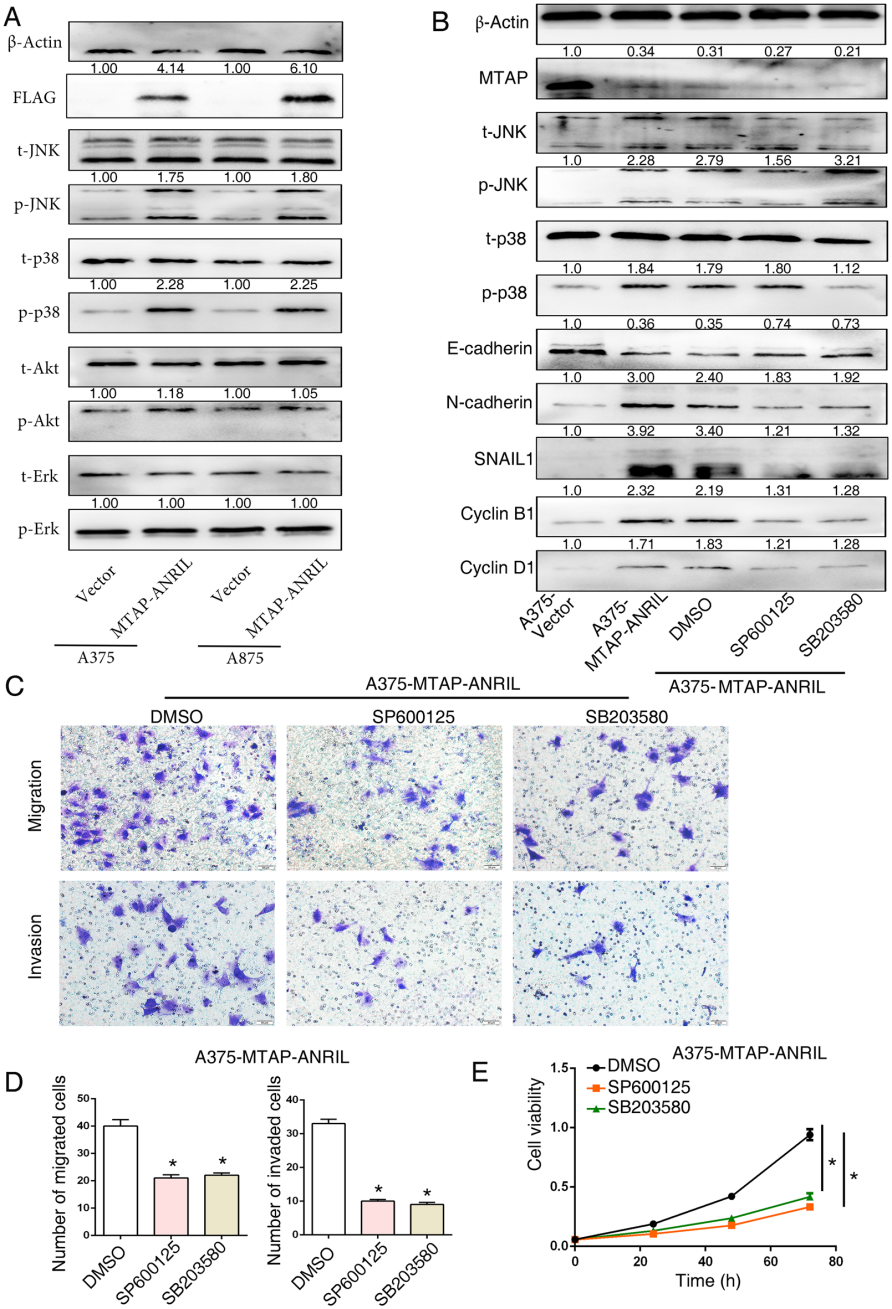
Overexpression of MTAP-ANRIL promoted melanoma migration, invasion and proliferation through activation of the JNK and p38 signaling pathways. (**A**) Western blot analysis of MTAP and phosphorylated and total Akt, ERK1/2, p38, and JNK after transinfected MTAP-ANRIL plasmid. (**B**) Western blot analysis of MTAP, E-cadherin, N-cadherin, SNAIL1, cyclin B1, cyclin D1, and phosphorylated and total p38, and JNK in A375 cells in the presence of the p38 inhibitor SB203580 (10 μM) and the JNK inhibitor SP600125 (10 μM). (**C**, **D**) Representative cell images and quantifications from transwell migration and invasion assays with A375 cells in the presence of the p38 inhibitor SB203580 (10 μM) and the JNK inhibitor SP600125 (10 μM). (**E**) CCK8 assays indicated that A375 cell proliferation was inhibited after treatment with the p38 inhibitor SB203580 (10 μM) and the JNK inhibitor SP600125 (10 μM). The data are presented as the mean ± SD values. *P < 0.01.

According to TCGA data for cutaneous melanoma, activations of RAF, RAS or loss of NF1, the three most frequently mutated genes, can subsequently activate the MAPK signaling pathway. In addition, higher frequencies of amplification and overexpression of AKT were detected in RAS, NF1 and triple wild-type melanomas^16^. Therefore, to determine which of these events contributed to the oncogenic effects of MTAP-ANRIL, we investigated the MAPK and Akt signaling pathways, which play key roles in tumor proliferation and metastasis. Overexpression of MTAP-ANRIL increased the phosphorylation of JNK and p38 but not Akt or ERK1/2 in melanoma cells (Fig. 4A).

To clarify whether MTAP-ANRIL induced SNAIL1, N-cadherin and Cyclin B1/Cyclin D1 expression via the JNK and p38 signaling pathways, MTAP-ANRIL melanoma cells were treated with a JNK inhibitor (SP600125) and a p38 inhibitor (SB203580). Inhibition of the JNK and p38 pathways in MTAP-ANRIL melanoma cells increased the expression level of E-cadherin but decreased the expression levels of SNAIL1, N-cadherin, cyclin B1 and cyclin D1 that were increased by MTAP-ANRIL (Fig. 4B).

Moreover, JNK and p38 pathway inhibitor treatment abolished MTAP-ANRIL-enhanced cell migration and invasion (Fig. 4C,D). Similarly, inactivation of the JNK and p38 signaling pathways inhibited the cell proliferation induced by MTAP-ANRIL overexpression (Fig. 4E). These findings indicated that MTAP-ANRIL inhibited E-cadherin expression and upregulated cyclin B1/cyclin D1 expression in melanoma cells via the JNK and p38 signaling pathways.

## Discussion

Although oncogenes such as RAF and RAS can be activated by somatic mutations, gene fusions may also play important roles in melanoma initiation and development^5,16^. To our knowledge, the MTAP-ANRIL gene fusions reported in our previous study are the most frequently occurring gene fusions in melanoma18. Here, we further observed that MTAP-ANRIL downregulated the expression of the wild-type tumor suppressor gene MTAP and promoted melanoma cell proliferation and metastasis.

Gene fusions usually occur between two coding genes, where coding regions or regulatory DNA sequences can be exchanged, and lead to the activation of functional units. In contrast, MTAP-ANRIL gene fusions occur between the housekeeping gene MTAP and the long noncoding RNA gene ANRIL and are consistent with recently reported pseudogene-associated and miRNA-convergent gene fusions in human cancers^29,30^. The MTAP-ANRIL fusion gene is translated into a truncated protein isoform of the tumor suppressor gene MTAP. Most gene truncations might be recognized as inactivation due to the lack of precise characterization of molecular effects. However, several truncated proteins have been proven to be functional^4,31,32^. For instance, truncated RUNX1 proteins translated from the RUNX1-TMEM48 fusion gene contribute to leukemogenesis by increasing proliferation and self-renewal through disruption of the wild-type RUNX1b gene^33^. Truncated BRCA1 proteins resulting from nonsense mutations can antagonize the function of the wild-type BRCA1 protein and actively promote oncogenesis as well as chemoresistance^34^. As mentioned above, truncated MTAP isoforms resulting from point mutations in MTAP exons can physically interact with wild-type MTAP and biologically active. Moreover, the overexpression of two new MTAP isoforms in patient-derived tissues suggested an oncogenic role for these newly discovered MTAP variants in hereditary malignant fibrous histiocytoma^20^.

EMT, a process by which epithelial cells transdifferentiate into motile mesenchymal cells, plays important roles in development, wound healing and stem cell behaviors and contributes pathologically to tumor metastasis^35^. During EMT, epithelial cancer cells lose epithelial differentiation and instead gain mesenchymal characteristics; thus, they can initiate early steps in metastasis, such as local tissue invasion and intravasation^2^. Although melanocytes are derived from the neural crest and differentiate into pigment-producing cells, they also possess some features of differentiated epithelial cells; moreover, EMT-like process promotes melanoma progression^2,36–38^. Mutations in RAC1 P29, the third most frequently mutated codon in human cutaneous melanoma, can activate the PAK, AKT and ARF/MRTF signaling pathways and lead to melanocytic mesenchymal phenotypic switching^39^. PTX3, a key factor in a subtype of invasive melanoma, can mediate the expression of the EMT transcription factor TWIST1 through a TLR4/MYD88/IKK/NF-κB signaling pathway and drive melanoma cell migration^40^. In the current study, we found that MTAP-ANRIL promoted melanoma cell metastasis by inducing EMT-like process through downregulating E-cadherin and upregulating N-cadherin. The expression of SNAIL1, which is a transcriptional factor of EMT, was increased when MTAP-ANRIL was up-regulated. In addition, we demonstrated that MTAP-ANRIL enhanced melanoma cell proliferation by upregulating the expression of the cell cycle regulators, cyclin B1 and cyclin D1. In parallel, we found that MTAP-ANRIL promoted melanoma cell metastasis and proliferation in vivo.

According to the molecular characteristics based on TCGA data for cutaneous melanoma, melanoma can be divided into four subtypes, namely, the BRAF, RAS, NF1 and triple- wild- type subtypes. BRAF mutations were detected in 52% and RAS mutations in 28% of melanomas, and BRAF and RAS mutations were mutually exclusive, meaning that collectively, BRAF and RAS mutations were detected in more than 80% of melanomas^16^. Moreover, activation of BRAF and RAS as well as NF1 can subsequently activate the MAPK signaling pathway, the most important pathway mediating melanoma metastasis^3,16^. Therefore, the RAF-RAS-MAPK signaling pathway predominantly promotes melanoma progression. Activation of three important MAPKs, namely, ERK, JNK and P38, mediates human tumor development^41^. Additionally, the AKT signaling pathway contributes to melanoma recurrence and plays an important role in melanoma progression^16^. To further investigate the mechanism of MTAP-ANRIL-induced EMT-like process and cell proliferation, we investigated the effect of MTAP-ANRIL on the MAPK and AKT signaling pathways. And found that MTAP-ANRIL activated the JNK and p38 signaling pathways without influencing both ERKs or Akt. Furthermore, we found that MTAP-ANRIL-enhanced melanoma cell migration and proliferation were abolished by pretreatment with JNK and p38 inhibitors. Collectively, these findings indicated that MTAP-ANRIL can promote melanoma cell migration and proliferation by activating the JNK and P38 signaling pathways.

In summary, our present study demonstrated that MTAP-ANRIL gene fusion can downregulate the expression of the wild-type tumor suppressor MTAP and promote melanoma cell migration via EMT-like process through the activation of JNK and p38 MAPKs. Gene fusions in solid tumors drive cancer development and progression, and act as useful biomarkers and even as promising targets for cancer treatment^4^. To our knowledge, this is the first study to show that MTAP-ANRIL plays oncogenic roles in melanoma. More studies should be performed to further confirm the functions of MTAP-ANRIL, which could be a molecular prognostic biomarker as well as a therapeutic target for melanoma.

## Supplementary Information

Supplementary Legends.

Supplementary Figure 1.

Supplementary Figures.

## Data Availability

All data generated or analysed during this study are included in this published article and its supplementary information files.

## References

[1] Bray F, Ferlay J, Soerjomataram I, Siegel RL, Torre LA, Jemal A (2018). Global cancer statistics 2018: GLOBOCAN estimates of incidence and mortality worldwide for 36 cancers in 185 countries. CA Cancer J. Clin..

[2] Damsky WE, Theodosakis N, Bosenberg M (2014). Melanoma metastasis: New concepts and evolving paradigms. Oncogene.

[3] Schadendorf D, van Akkooi ACJ, Berking C, Griewank KG, Gutzmer R, Hauschild A, Stang A, Roesch A, Ugurel S (2018). Melanoma. Lancet.

[4] Mertens F, Johansson B, Fioretos T, Mitelman F (2015). The emerging complexity of gene fusions in cancer. Nat. Rev. Cancer.

[5] Mitelman F, Johansson B, Mertens F (2004). Fusion genes and rearranged genes as a linear function of chromosome aberrations in cancer. Nat. Genet..

[6] Kumar-Sinha C, Tomlins SA, Chinnaiyan AM (2008). Recurrent gene fusions in prostate cancer. Nat. Rev. Cancer.

[7] Tomlins SA, Rhodes DR, Perner S, Dhanasekaran SM, Mehra R, Sun XW, Varambally S, Cao X, Tchinda J, Kuefer R (2005). Recurrent fusion of TMPRSS2 and ETS transcription factor genes in prostate cancer. Science.

[8] Soda M, Choi YL, Enomoto M, Takada S, Yamashita Y, Ishikawa S, Fujiwara S, Watanabe H, Kurashina K, Hatanaka H (2007). Identification of the transforming EML4-ALK fusion gene in non-small-cell lung cancer. Nature.

[9] Seshagiri S, Stawiski EW, Durinck S, Modrusan Z, Storm EE, Conboy CB, Chaudhuri S, Guan Y, Janakiraman V, Jaiswal BS (2012). Recurrent R-spondin fusions in colon cancer. Nature.

[10] Lipson D, Capelletti M, Yelensky R, Otto G, Parker A, Jarosz M, Curran JA, Balasubramanian S, Bloom T, Brennan KW (2012). Identification of new ALK and RET gene fusions from colorectal and lung cancer biopsies. Nat. Med..

[11] Bass AJ, Lawrence MS, Brace LE, Ramos AH, Drier Y, Cibulskis K, Sougnez C, Voet D, Saksena G, Sivachenko A (2011). Genomic sequencing of colorectal adenocarcinomas identifies a recurrent VTI1A-TCF7L2 fusion. Nat. Genet..

[12] Tao J, Deng NT, Ramnarayanan K, Huang B, Oh HK, Leong SH, Lim SS, Tan IB, Ooi CH, Wu J (2011). CD44-SLC1A2 gene fusions in gastric cancer. Sci. Transl. Med..

[13] Robinson DR, Kalyana-Sundaram S, Wu YM, Shankar S, Cao X, Ateeq B, Asangani IA, Iyer M, Maher CA, Grasso CS (2011). Functionally recurrent rearrangements of the MAST kinase and Notch gene families in breast cancer. Nat. Med..

[14] Palanisamy N, Ateeq B, Kalyana-Sundaram S, Pflueger D, Ramnarayanan K, Shankar S, Han B, Cao Q, Cao X, Suleman K (2010). Rearrangements of the RAF kinase pathway in prostate cancer, gastric cancer and melanoma. Nat. Med..

[15] Yeh I, Botton T, Talevich E, Shain AH, Sparatta AJ, de la Fouchardiere A, Mully TW, North JP, Garrido MC, Gagnon A (2015). Activating MET kinase rearrangements in melanoma and Spitz tumours. Nat. Commun..

[16] Cancer Genome Atlas N (2015). Genomic classification of cutaneous melanoma. Cell.

[17] Tate JG, Bamford S, Jubb HC, Sondka Z, Beare DM, Bindal N, Boutselakis H, Cole CG, Creatore C, Dawson E (2019). COSMIC: The catalogue of somatic mutations in cancer. Nucl. Acids Res..

[18] Xie H, Rachakonda PS, Heidenreich B, Nagore E, Sucker A, Hemminki K, Schadendorf D, Kumar R (2016). Mapping of deletion breakpoints at the CDKN2A locus in melanoma: detection of MTAP-ANRIL fusion transcripts. Oncotarget.

[19] Walter W, Shahswar R, Stengel A, Meggendorfer M, Kern W, Haferlach T, Haferlach C (2021). Clinical application of whole transcriptome sequencing for the classification of patients with acute lymphoblastic leukemia. BMC Cancer.

[20] Camacho-Vanegas O, Camacho SC, Till J, Miranda-Lorenzo I, Terzo E, Ramirez MC, Schramm V, Cordovano G, Watts G, Mehta S (2012). Primate genome gain and loss: A bone dysplasia, muscular dystrophy, and bone cancer syndrome resulting from mutated retroviral-derived MTAP transcripts. Am. J. Hum. Genet..

[21] Christopher SA, Diegelman P, Porter CW, Kruger WD (2002). Methylthioadenosine phosphorylase, a gene frequently codeleted with p16(cdkN2a/ARF), acts as a tumor suppressor in a breast cancer cell line. Cancer Res..

[22] Tang B, Kadariya Y, Chen Y, Slifker M, Kruger WD (2014). Expression of MTAP inhibits tumor-related phenotypes in HT1080 cells via a mechanism unrelated to its enzymatic function. G3 Bethesda.

[23] Kryukov GV, Wilson FH, Ruth JR, Paulk J, Tsherniak A, Marlow SE, Vazquez F, Weir BA, Fitzgerald ME, Tanaka M (2016). MTAP deletion confers enhanced dependency on the PRMT5 arginine methyltransferase in cancer cells. Science.

[24] Mavrakis KJ, McDonald ER, Schlabach MR, Billy E, Hoffman GR, deWeck A, Ruddy DA, Venkatesan K, Yu J, McAllister G (2016). Disordered methionine metabolism in MTAP/CDKN2A-deleted cancers leads to dependence on PRMT5. Science.

[25] Marjon K, Cameron MJ, Quang P, Clasquin MF, Mandley E, Kunii K, McVay M, Choe S, Kernytsky A, Gross S (2016). MTAP deletions in cancer create vulnerability to targeting of the MAT2A/PRMT5/RIOK1 axis. Cell Rep..

[26] Cheng XY, Liu Z, Shang L, Cai HQ, Zhang Y, Cai Y, Xu X, Hao JJ, Wang MR (2017). Deletion and downregulation of MTAP contribute to the motility of esophageal squamous carcinoma cells. Onco Targets Ther..

[27] Holdt LM, Hoffmann S, Sass K, Langenberger D, Scholz M, Krohn K, Finstermeier K, Stahringer A, Wilfert W, Beutner F (2013). Alu elements in ANRIL non-coding RNA at chromosome 9p21 modulate atherogenic cell functions through trans-regulation of gene networks. PLoS Genet.

[28] Barbero G, Castro MV, Villanueva MB, Quezada MJ, Fernandez NB, DeMorrow S, Lopez-Bergami P (2019). An autocrine Wnt5a loop promotes NF-kappaB pathway activation and cytokine/chemokine secretion in melanoma. Cells.

[29] Persson H, Sokilde R, Hakkinen J, Pirona AC, Vallon-Christersson J, Kvist A, Mertens F, Borg A, Mitelman F, Hoglund M (2017). Frequent miRNA-convergent fusion gene events in breast cancer. Nat Commun.

[30] Chakravarthi BV, Dedigama-Arachchige P, Carskadon S, Sundaram SK, Li J, Wu KH, Chandrashekar DS, Peabody JO, Stricker H, Hwang C (2019). Pseudogene associated recurrent gene fusion in prostate cancer. Neoplasia.

[31] Rivas MA, Pirinen M, Conrad DF, Lek M, Tsang EK, Karczewski KJ, Maller JB, Kukurba KR, DeLuca DS, Fromer M (2015). Human genomics. Effect of predicted protein-truncating genetic variants on the human transcriptome. Science.

[32] Holbrook JA, Neu-Yilik G, Hentze MW, Kulozik AE (2004). Nonsense-mediated decay approaches the clinic. Nat. Genet..

[33] Rodriguez-Perales S, Torres-Ruiz R, Suela J, Acquadro F, Martin MC, Yebra E, Ramirez JC, Alvarez S, Cigudosa JC (2015). Truncated RUNX1 protein generated by a novel t(1;21)(p32;q22) chromosomal translocation impairs the proliferation and differentiation of human hematopoietic progenitors. Oncogene.

[34] Fan S, Yuan R, Ma YX, Meng Q, Goldberg ID, Rosen EM (2001). Mutant BRCA1 genes antagonize phenotype of wild-type BRCA1. Oncogene.

[35] Lamouille S, Xu J, Derynck R (2014). Molecular mechanisms of epithelial-mesenchymal transition. Nat. Rev. Mol. Cell Biol..

[36] Weiss MB, Abel EV, Mayberry MM, Basile KJ, Berger AC, Aplin AE (2012). TWIST1 is an ERK1/2 effector that promotes invasion and regulates MMP-1 expression in human melanoma cells. Cancer Res..

[37] Caramel J, Papadogeorgakis E, Hill L, Browne GJ, Richard G, Wierinckx A, Saldanha G, Osborne J, Hutchinson P, Tse G (2013). A switch in the expression of embryonic EMT-inducers drives the development of malignant melanoma. Cancer Cell.

[38] Pedri D, Karras P, Landeloos E, Marine JC, Rambow F (2021). Epithelial-to-mesenchymal-like transition events in melanoma. FEBS J..

[39] Lionarons DA, Hancock DC, Rana S, East P, Moore C, Murillo MM, Carvalho J, Spencer-Dene B, Herbert E, Stamp G (2019). RAC1(P29S) induces a mesenchymal phenotypic switch via serum response factor to promote melanoma development and therapy resistance. Cancer Cell.

[40] Rathore M, Girard C, Ohanna M, Tichet M, Ben Jouira R, Garcia E, Larbret F, Gesson M, Audebert S, Lacour JP (2019). Cancer cell-derived long pentraxin 3 (PTX3) promotes melanoma migration through a toll-like receptor 4 (TLR4)/NF-kappaB signaling pathway. Oncogene.

[41] Wagner EF, Nebreda AR (2009). Signal integration by JNK and p38 MAPK pathways in cancer development. Nat. Rev. Cancer.

