# Supplementary material for: MTAP-ANRIL gene fusion promotes melanoma epithelial-mesenchymal transition-like process by activating the JNK and p38 signaling pathways
Source: Sci Rep. 2023 Jun 5;13:9073. doi: 10.1038/s41598-023-36404-w (PMC10241944; doi:10.1038/s41598-023-36404-w)
Supplement: Supplementary file 1 — Supplementary Legends. [file 41598_2023_36404_MOESM1_ESM.docx]

**Supplementary Figure S1.**

(A) The mRNA level of MTAP were detected in the pan-cancer from HPA database.

(B) The mRNA level of MTAP in the melanoma by HPA database.

(C) Typical image of immunohistochemistry-stained melanoma tissues by HPA database.
