# Supplementary figures and images for: MTAP-ANRIL gene fusion promotes melanoma epithelial-mesenchymal transition-like process by activating the JNK and p38 signaling pathways
Source: Sci Rep. 2023 Jun 5;13:9073. doi: 10.1038/s41598-023-36404-w (PMC10241944; doi:10.1038/s41598-023-36404-w)

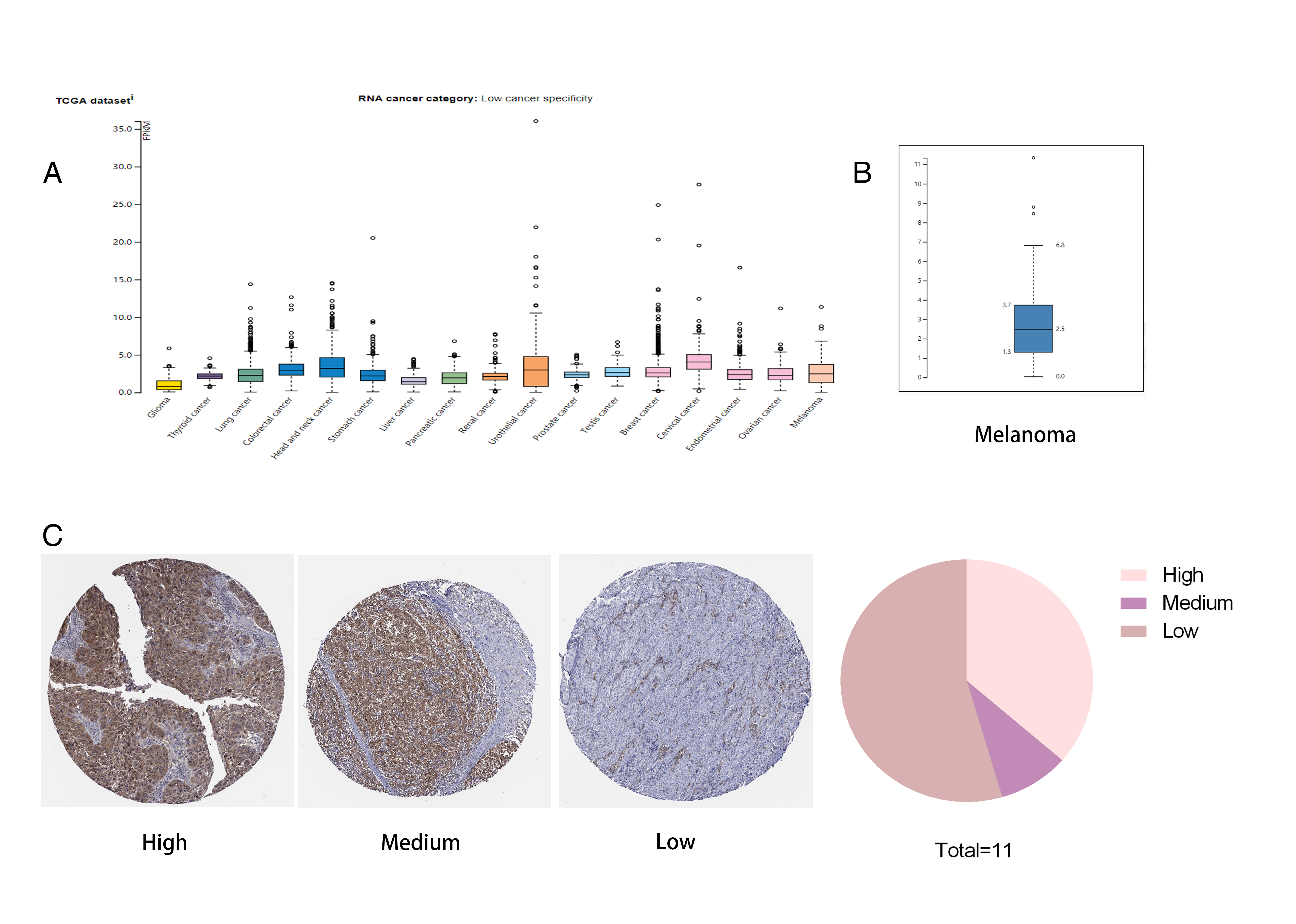

Supplement: Supplementary file 2 — Supplementary Figure 1. [file 41598_2023_36404_MOESM2_ESM.tif]
