# Supplementary material for: MTAP-ANRIL gene fusion promotes melanoma epithelial-mesenchymal transition-like process by activating the JNK and p38 signaling pathways
Source: Sci Rep. 2023 Jun 5;13:9073. doi: 10.1038/s41598-023-36404-w (PMC10241944; doi:10.1038/s41598-023-36404-w)

Due to the early experimental habits and limited funds, we will apply membrane of different lengths on a piece of gel to save space, so the strips will be cut. But we can guarantee that the results are real and valid. Here, we have provided the images of all blots as they are, with membrane edges visible.

Figure1
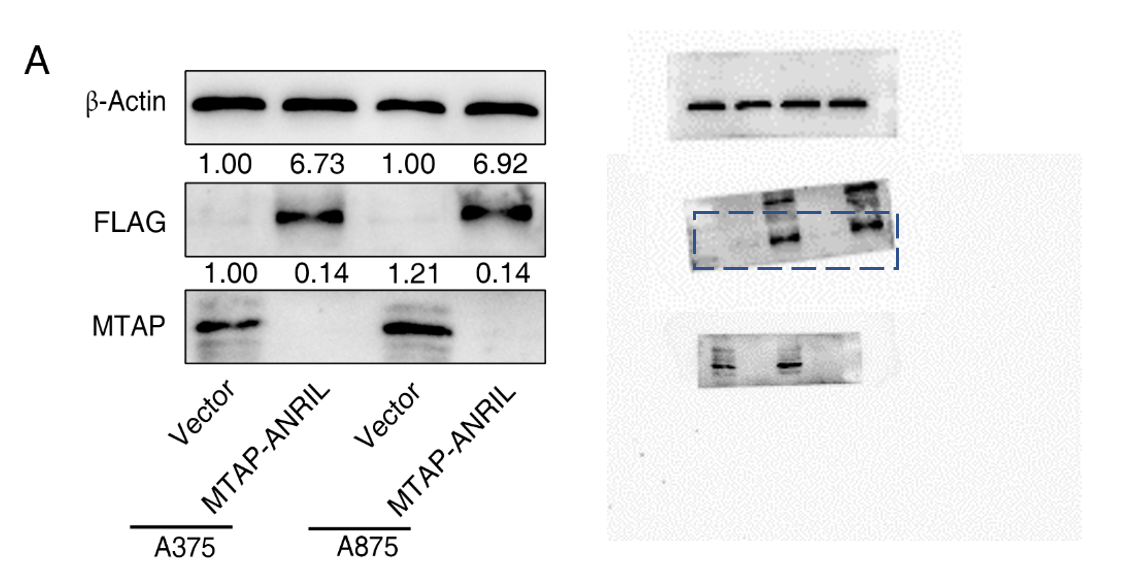


Figure3


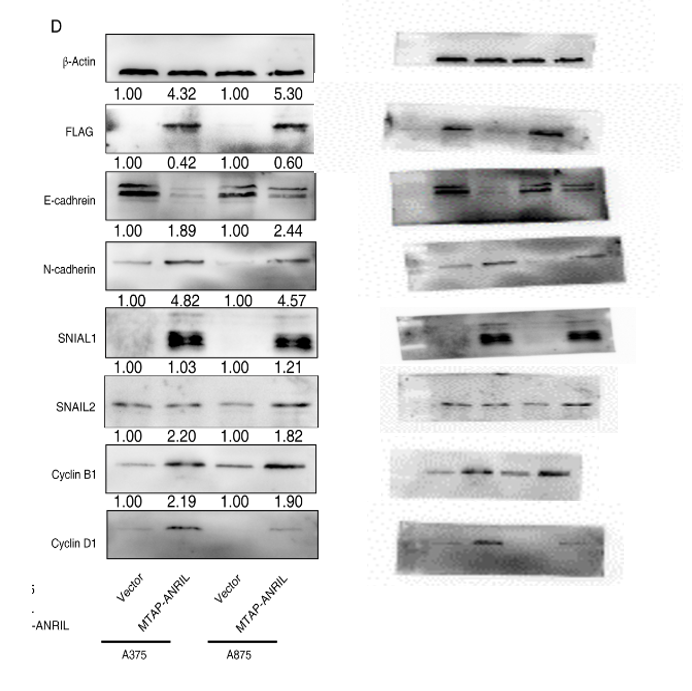


Figure4


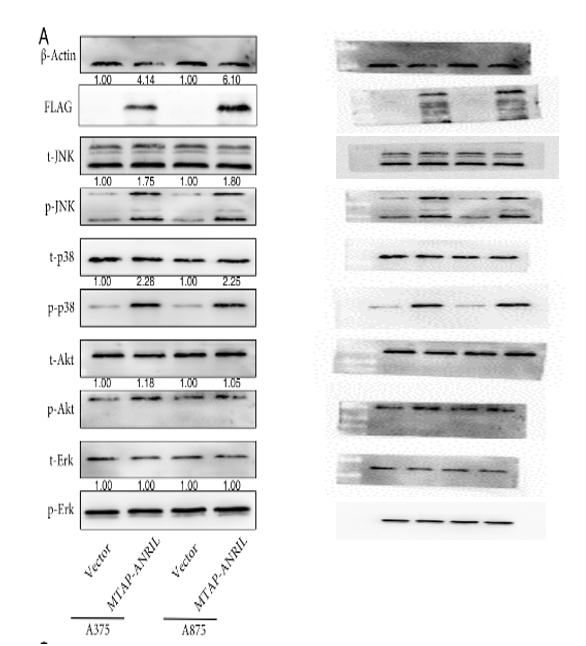


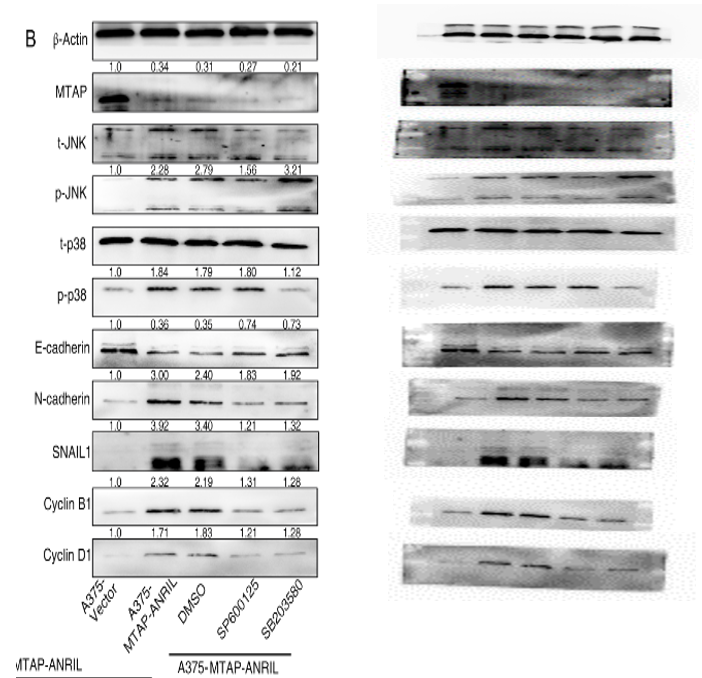

Supplement: Supplementary file 3 — Supplementary Figures. [file 41598_2023_36404_MOESM3_ESM.docx]
